# Systematic review of cognitive impairment and brain insult after mechanical ventilation

**DOI:** 10.1186/s13054-021-03521-9

**Published:** 2021-03-10

**Authors:** Thiago G. Bassi, Elizabeth C. Rohrs, Steven C. Reynolds

**Affiliations:** 1 Simon Fraser University, Burnaby, Canada; 2 Lungpacer Medical Inc, Vancouver, Canada; 3 Royal Columbian Hospital, Fraser Health Authority, 260 Sherbrooke Street, New Westminster, BC V3L 3M2 Canada

**Keywords:** Ventilators, Mechanical, Brain injuries, Delirium, Apoptosis, Cognitive impairment

## Abstract

We conducted a systematic review following the PRISMA protocol primarily to identify publications that assessed any links between mechanical ventilation (MV) and either cognitive impairment or brain insult, independent of underlying medical conditions. Secondary objectives were to identify possible gaps in the literature that can be used to inform future studies and move toward a better understanding of this complex problem. The preclinical literature suggests that MV is associated with neuroinflammation, cognitive impairment, and brain insult, reporting higher neuroinflammatory markers, greater evidence of brain injury markers, and lower cognitive scores in subjects that were ventilated longer, compared to those ventilated less, and to never-ventilated subjects. The clinical literature suggests an association between MV and delirium, and that delirium in mechanically ventilated patients may be associated with greater likelihood of long-term cognitive impairment; our systematic review found no clinical study that demonstrated a causal link between MV, cognitive dysfunction, and brain insult. More studies should be designed to investigate ventilation-induced brain injury pathways as well as any causative linkage between MV, cognitive impairment, and brain insult.

**Supplementary Information:**

The online version contains supplementary material available at 10.1186/s13054-021-03521-9.

## Introduction

Mechanical ventilation (MV) is considered essential in the Intensive Care Unit (ICU) [1]. While it is undeniable that MV is a crucial life-support tool, it may also cause injury to distal organs, such as the lungs, diaphragm, and brain [2, 3]. Ventilation-induced brain injury (VIBI) is well known in neonatology, as a consequence of either hyperoxia or the use of intermittent positive pressure ventilation [4]; in adult patients, the existence of VIBI is still unknown. Preclinical experiments have, however, shown lower cognitive scores in subjects ventilated longer, and that these subjects had greater levels of brain insult, neuroinflammation, and neuronal apoptosis than subjects either mechanically ventilated less, or spontaneously breathing [5, 6]. Currently, direct links between MV, delirium, cognitive impairment, and neuroinflammation have not been established in the literature.

Delirium is a complex disturbance of consciousness, characterized by acute changes in cognition, a direct consequence of a medical condition, medical treatment, or intoxicating substance [7]. Pathophysiologically, some authors have classified the mechanism that triggers delirium into two distinct categories, direct brain insult (such as hemorrhagic stroke), and aberrant stress response (such as systemic stress induced by MV, sepsis, septic shock, systemic inflammation post-surgery, etc.) [8]. Regardless of the mechanism that triggers delirium, it is postulated that delirium is a result of an imbalance in neurotransmitters, specifically acetylcholine and dopamine, impairing the connection among several brain areas [8–11]. Taking as an example a case–control post-mortem study of deceased ICU patients without direct brain injury, higher levels of inflammatory cells were reported in the hippocampi of deceased patients with delirium than in patients without delirium [5]. This indicates that when neuroinflammation is triggered, by direct brain insult, aberrant systemic stress response, or some other mechanism, it may be associated with cognitive dysfunction [5, 12, 13]. However, it is likely that there are many factors beyond neuroinflammation that can contribute to cognitive impairment in the ICU, such as medications, immobility, overload of sensory input and lack of adequate sleep [7–10].

We sought to explore the current knowledge in the literature regarding MV, delirium, cognitive impairment, and neuroinflammation, through a systematic review. The primary objective of this systematic review was to identify published papers that assess any link between MV and either cognitive impairment or brain insult, independent of underlying medical conditions. Our secondary objective was to identify possible gaps in the literature that can inform the design of future studies for a better understanding of this complex problem.

## Methodology

This study was conducted following the Preferred Reporting Items for Systematic Review and Meta-Analysis (PRISMA) protocol. Searches were performed by a librarian at the Health Sciences Library at Fraser Health Authority, Royal Columbian Hospital (New Westminster, Canada). Searches were conducted using the following sources: Medline (1946-present), EMBASE (1974-present), Cochrane Database of Systematic Reviews, Cochrane Central Register of Controlled Trials, Cochrane Methodology Register, and the Database of Abstracts of Reviews and Effects (DARE).

**Fig. 1:**
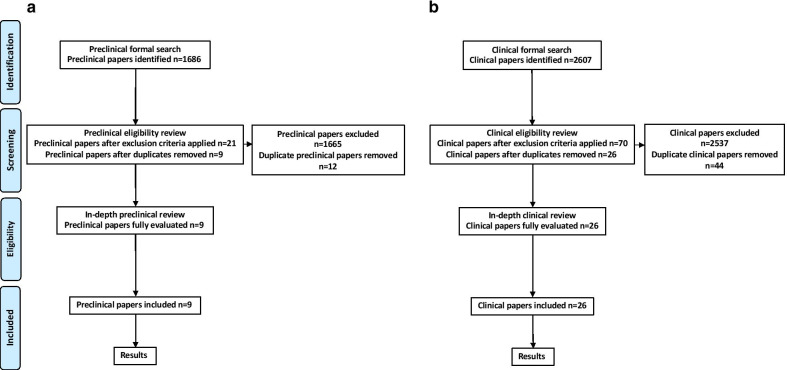
Systematic review process and results. **a** Review process for the preclinical papers. **b** Review process for the clinical papers

Search strategies were developed based on the search interface, to ensure an appropriate balance between search sensitivity and specificity. The searches were also stratified into two separate concepts, with one search conducted in each database focused on ‘preclinical’ articles concerning any link between MV and brain insult (Fig. 1a), and a second search focused on ‘clinical’ articles concerning any link between MV and either cognitive impairment or delirium during the hospital stay and after hospital discharge (Fig. 1b). The preclinical papers were used for consideration of putative mechanisms for brain insult after MV and to identify gaps in the preclinical literature. Articles were limited to prospective and retrospective studies published in English. Keyword, adjacency, wildcard, and subject heading searching were employed in all search strategies to maximize the sensitivity of the search, while publication limits, specific clinical terms, and variants of these were used to increase the specificity of the search results.

Formal searches for articles were run using pre-established keywords (see Additional file 1: Table A), all of which were conducted on January 28, 2020. Subsequently, a screening review of all the articles identified in the formal searches was performed. During the screening review, the abstract of each article was reviewed by the lead author (TGB) to identify articles that clearly met the predetermined exclusion criteria of our study protocol (see Additional file 1: Table B). Duplicate articles were eliminated as part of the screening review. Articles not eliminated during the screening review then underwent an in-depth review. The full manuscripts selected were evaluated independently by two investigators (TGB and ECR) to reduce the risk of individual bias. The two reviewers (TGB and ECR) used the Downs and Black checklist to assess the quality of each included full manuscript, to compare and reconcile independent evaluations. In the event of significant score discrepancies, a third reviewer (SCR) independently evaluated the paper and served as the adjudicator.

The clinical articles were grouped into three subgroups: papers that reported MV as an independent variable increasing the likelihood for delirium; papers that reported delirium as an independent variable increasing the likelihood for prolonged MV; papers that reported delirium in mechanically ventilated patients increasing the likelihood for long-term cognitive impairment. Odds ratios (OR) were used to calculate the weight and heterogeneity of the manuscripts, using inverse covariance with a random-effects model. Where it was not provided, OR was calculated utilizing data in the papers. *P* value < 0.1 for the chi-square test was considered significant. Heterogeneity was evaluated using Higgins metric (*I*^2^), where *I*^2^ > 75% was considered significant heterogeneity, *I*^2^ of 40–74% was considered moderate heterogeneity, and *I*^2^ < 39% was considered no heterogeneity. All statistical analyses were performed using Review Manager software (RevMan, Version 5.4.1, The Cochrane Collaboration, 2020).

## Results

Results from our preclinical and clinical systematic reviews are available in Tables 1 and 2, respectively. Nine preclinical publications and 26 clinical publications were identified. The papers reviewed were produced in five continents: North America 9 (9 clinical, 0 preclinical), South America 3 (3 clinical, 0 preclinical), Europe 12 (5 clinical, 7 preclinical), Asia 9 (7 clinical, 2 preclinical), and Oceania 2 (2 clinical, 0 preclinical).

**Table 1:** Summary of preclinical publications reviewed

Publication	Population	Mechanical ventilation/experimental model	Neuro-inflammation/cellular apoptosis observed after mechanical ventilation	Low cognitive scores measured after mechanical ventilation	Brain area(s) studied
2005, Fries et al. [14]	Pigs (*n* = 14)	Yes/tidal volume 10 ml/kg, lung injury (by reduced inspired oxygen and by bronchoalveolar lavage)	Yes	–	Hippocampus
2011, Quilez et al. [15]	Mice (*n* = 24)	Yes/low tidal volume (8 ml/kg), high tidal volume (30 ml/kg), and spontaneously breathing	Yes	–	Hippocampus, retrosplenial cortex, thalamus, central amygdala, paraventricular nuclei, and supraoptic nuclei
2011, Bickenbach et al. [16]	Pigs (*n* = 10)	Yes/tidal volume 10 ml/kg, lung injury (by oleic acid and by bronchoalveolar lavage)	Yes	Yes	Hippocampus
2013, Gonzalez-Lopez et al. [4]	Mice (*n* = 127)	Yes/low peak inspiratory pressure (12 cmH_2_O) and high peak inspiratory pressure (20 cmH_2_O)	Yes	–	Hippocampus
2015, Chen et al. [5]	Mice (*n* = 86)	Yes/peak inspiratory pressure (15 cmH_2_O) (1 h, 3 h and 6 h), and spontaneously breathing	Yes	Yes	Hippocampus
2016, Chen et al. [6]	Mice (*n* = 72)	Yes/peak inspiratory pressure (15 cmH_2_O) (1 h, 3 h and 6 h), and spontaneously breathing	Yes	Yes	Hippocampus
2018, Kamuf et al. [13]	Pigs (*n* = 20)	Yes/tidal volume 7 ml/kg, lung injury (by oleic acid and by bronchoalveolar lavage)	Yes	–	Hippocampus
2019, Lopez-Aguilar et al. [17]	Pigs (*n* = 17)	Yes/tidal volume 10 ml/kg, three different head positions (+ 30°, + 5°, − 30°)	Yes	–	Hippocampus
2019, Gonzalez-Lopez et al. [18]	Mice (*n* = 32)	Yes/high tidal volume (20–30 ml/kg) and spontaneously breathing	Yes	–	Hippocampus

**Table 2:** Summary of clinical publications reviewed

Publication	Study type	Number of patients	Summary of participants	Outcomes analyzed	
				Delirium	Long-term cognitive impairment
2002, Granberg et al. [19]	Prospective cohort study	19	Mechanically ventilated ICU patients	Yes	**–**
2004, Ely et al. [20]	Prospective cohort study	275	Mechanically ventilated ICU patients	Yes	Yes
2006, Peterson et al. [21]	Prospective cohort study	375	ICU patients	Yes	**–**
2007, Balas et al. [22]	Prospective cohort study	114	ICU patients	Yes	**–**
2008, Lin et al. [23]	Prospective cohort study	143	Mechanically ventilated ICU patients	Yes	**–**
2009, Rompaey et al. [24]	Prospective cohort study	523	ICU patients	Yes	**–**
2010, Girard et al. [25]	Prospective cohort study	126	Mechanically ventilated ICU patients	Yes	Yes
2010, Tsuruta et al. [26]	Prospective cohort study	172	ICU patients	Yes	**–**
2010, Shehabi et al. [27]	Prospective cohort study	354	Mechanically ventilated ICU patients	Yes	**–**
2012, Sharma et al. [28]	Prospective cohort study	140	ICU patients	Yes	**–**
2013, Haas et al. [29]	Prospective cohort study	1216	ICU patients	Yes	Yes
2013, Norkiene et al. [30]	Prospective cohort study	87	Cardiovascular surgery patients	Yes	**–**
2014, Brummel et al. [31]	Prospective cohort study	126	Mechanically ventilated ICU patients	Yes	Yes
2014, Tsuruta et al. [32]	Prospective cohort study	180	Mechanically ventilated ICU patients	Yes	Yes
2014, Connor et al. [33]	Prospective cohort study	80	Mechanically ventilated ICU patients	Yes	**–**
2015, Mehta et al. [34]	Prospective cohort study	430	Mechanically ventilated ICU patients	Yes	Yes
2015, Hsieh et al. [35]	Prospective cohort study	564	Mechanically ventilated ICU patients	Yes	**–**
2016, Almeida et al. [36]	Prospective cohort study	113	ICU patients	Yes	**–**
2017, Chen et al. [37]	Prospective cohort study	620	ICU patients	Yes	**–**
2017, Mesa et al. [38]	Prospective cohort study	230	Mechanically ventilated ICU patients	Yes	**–**
2017, Rueden et al. [39]	Prospective cohort study	215	Trauma patients	Yes	**–**
2018, Shehabi et al. [40]	Prospective cohort multicenter study	710	Mechanically ventilated ICU patients	Yes	**–**
2018, Singh et al. [41]	Retrospective cohort study	67,333	ICU patients	Yes	**–**
2018, Sanchez-Hurtado et al. [42]	Prospective cohort study	109	ICU/cancer patients	Yes	**–**
2018, Mitchell et al. [43]	Prospective cohort study	148	Mechanically ventilated ICU patients	Yes	Yes
2019, Torres-Contreras et al. [44]	Prospective cohort study	134	ICU patients	Yes	**–**

Papers were scored according to the Downs and Black checklist. Of the 35 papers selected, 0 scored ‘excellent,’ 15 (43%) scored ‘good’ (9 clinical, 6 preclinical), 18 (51%) scored ‘fair’ (clinical 15, preclinical 3), and 2 (6%) scored ‘poor’ (both clinical).

All nine preclinical papers measured neuroinflammation, and seven also used brain cellular apoptosis after MV as an outcome (see Table 1). Neuroinflammation was indicated by the elevated presence of microglia, elevated presence of reactive astrocytes, or elevated presence of inflammatory markers. Brain cellular apoptosis was demonstrated by terminal deoxynucleotidyl transferase dUTP nick end labeling (TUNEL) positive cells, by phosphorylation of glycogen synthetase kinase 3b (GSK3b), or by cleavage of poly-adenosine-diphosphate-ribose polymerase-1 (PARP-1). One paper used S100 serum concentration to demonstrate brain insult. Three preclinical papers evaluated cognition after MV, all showing lower cognitive scores in subjects after mechanical ventilation. These three papers used as a measurement of cognitive function either a fear-conditioning test to quantify freezing time, or a validated porcine neurological deficit score.

All 26 clinical papers evaluated delirium during hospitalization as either a primary or secondary variable of interest (Table 2). The most common population studied was ICU patients (24 publications), followed by cardiovascular surgical patients (one publication) and trauma patients (one publication) (Table 2). Thirteen papers included exclusively mechanically ventilated ICU patients in their studies (Table 2). Duration of MV, greater administration of sedative drugs, age > 65, physical immobility, physical restraint, low APACHE II score, sepsis, hypertension, low level of hemoglobin at hospital admission, smoking, alcohol consumption (> 2 drinks daily), and low albumin concentration at ICU admission were risk factors identified either for delirium during hospitalization or for long-term cognitive impairment after hospital discharge.

**Fig. 2:**
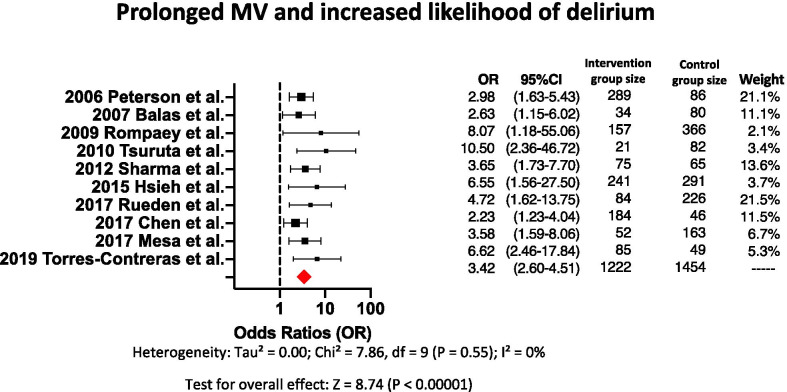
Forest plot showing the odds ratios for duration of MV as an independent variable associated with increased likelihood of delirium. The size of each black dot corresponds to the weight effect of the study in the meta-analysis. Red diamond represents the pooled odds ratio

Twelve clinical papers found that duration of MV is an independent variable associated with a greater likelihood of patients developing delirium during hospitalization. Ten of these twelve papers reported odds ratios ranging from 2.23 to 10.50, with a pooled odds ratio of 3.42 (Fig. 2). No heterogeneity between the papers was observed with *p* = 0.55 for Chi-square, and an *I*^2^ of 0%. One paper that included only mechanically ventilated patients reported that delirium was diagnosed in 68% of the patients studied; in those patients diagnosed with delirium, median duration of delirium was 1 day (IQR 1–2), and median day of occurrence of delirium was day 5 of MV (IQR 3–7); the authors concluded that prolonged MV is associated with greater likelihood of delirium during hospitalization [43] . Another paper reported that in cancer patients, time of MV increased the likelihood for delirium with an OR of 1.06. This paper reported an average of 8 days of MV for patients with delirium and 2 days of MV for patients without delirium.

**Fig. 3:**
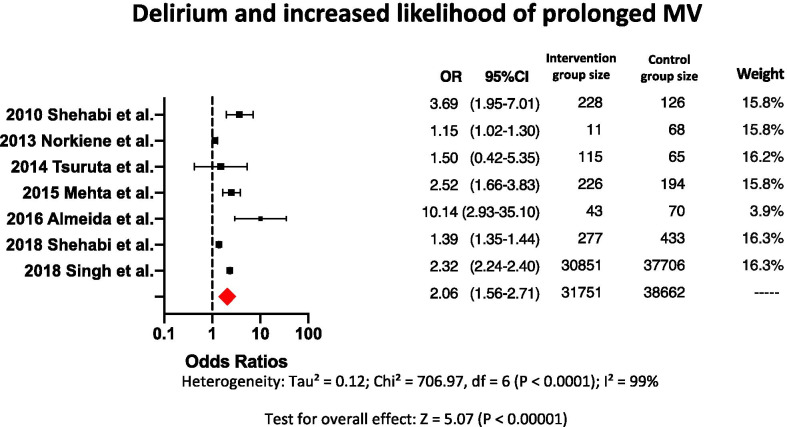
Forest plot showing the odds ratios for delirium as an independent variable associated with increased likelihood of prolonged MV. The size of each black dot corresponds to the weight effect of the study in the meta-analysis. Red diamond represents the pooled odds ratio

Nine clinical papers found that delirium during hospitalization is an independent variable associated with a greater likelihood for a longer duration of MV in ICU patients. Seven of these nine papers reported odds ratios ranging from 1.15 to 10.14 with a pooled odds ratio of 2.06 (Fig. 3). Three of these seven papers did not originally report odds ratio; odds ratios were calculated from data in these three papers. The heterogeneity between the papers was considered substantial with *p* < 0.0001 for Chi-square and an *I*^2^ of 99%. Of the nine papers, one reported that patients with severe delirium were mechanically ventilated for a median of 222 h (IQR 106–384), patients with moderate delirium were mechanically ventilated for a median of 24 h (IQR 12–124), and patients with no delirium were mechanically ventilated for a median of 18 h (IQR 12–41), with statistically significant differences between the groups, *p* = 0.001 [22]. One of the nine papers reported that patients with delirium during hospitalization were mechanically ventilated longer than patients without delirium during hospitalization (19.5 days, SD 15.8 vs. 9.3 days, SD 8.8, respectively, *p* = 0.003) [17].

**Fig. 4:**
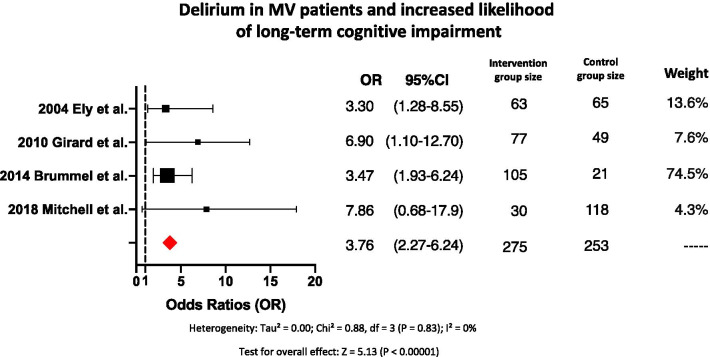
Forest plot showing the odds ratios for delirium during MV as an independent variable associated with increased likelihood of long-term cognitive impairment. The size of each black dot corresponds to the weight effect of the study in the meta-analysis. Red diamond represents the pooled odds ratio

Five clinical papers found delirium as a predictor of a greater likelihood for chronic cognitive impairment. All five papers included exclusively mechanically ventilated patients. These five papers reported odds ratios ranging from 3.30 to 7.86 with a pooled odds ratio of 3.76 (Fig. 4). One of these five papers did not originally report odds ratio; the odds ratio was calculated from data in that paper. No heterogeneity between the papers was observed, with *p* = 0.83 for Chi-square and an *I*^2^ of 0%.

Another paper reported that more than 8 days of MV increased the likelihood for long-term cognitive impairment two years after hospitalization, with a risk ratio of 1.48 [24].

## Discussion

Our preclinical search found evidence that MV is a contributing factor that can induce brain insult, either by inducing systemic inflammation or by changing the vagal signal, associated with neuroinflammation and neuronal death [3, 5, 6, 13–18]. Moreover, all preclinical studies reported in our systematic review found brain insult after MV [3, 5, 6, 13–18]; three preclinical studies found an association between the brain insult and worse cognitive scores in prolonged mechanically ventilated subjects in comparison with either never-ventilated subjects or short-term mechanically ventilated subjects [3, 5, 6]. Although these studies were not able to entirely control for factors that co-varied with MV, such as sedation or immobility, there is a consistent signal across all studies showing an association between MV and brain insult. High levels of pro-apoptotic proteins and elevated levels of inflammatory cells in the brain after MV were reported in nine preclinical papers identified in our systematic review (see Table 1) [3, 5, 6, 13–18].

### Preclinical considerations regarding putative mechanisms for cognitive impairment after MV

According to our preclinical search, the mechanism of action for brain insult after MV has two postulated pathways, inflammation and neural signaling [3, 5, 6, 13–18].

#### Inflammatory pathway

Six preclinical papers investigated the inflammatory pathway for brain insult after MV [5, 6, 13–15]. In the postulated inflammatory pathway for VIBI, MV creates a pro-inflammatory systemic state that triggers neuroinflammation in the brain [5, 6, 15]. Three preclinical papers found that subjects mechanically ventilated for a longer duration (6 h) had greater serum inflammatory markers and greater neuroinflammation either than subjects ventilated for a shorter duration (1 h) or than subjects that were never ventilated [5, 6, 15]. One study demonstrated that the activation of pulmonary toll-like receptor-4 was responsible for the initiation of the inflammatory cascade since toll-like receptor-4 knockout subjects did not demonstrate the neuroinflammatory effects after MV, even when ventilated longer (6 h) [6]. This same study showed that toll-like receptor-4 knockout subjects after six hours of MV had cognitive scores (freezing time and locomotor activity) similar to the never-ventilated group [6]. The inflammatory hypothesis was challenged by one study that investigated the levels of inflammatory and apoptotic markers in the hippocampus after inducing lung injury [13]. This study showed that mechanically ventilated pigs without lung injury and mechanically ventilated pigs with lung injury showed similar levels of inflammatory and apoptotic brain markers, thereby concluding that the inflammatory process induced by lung injury was not the factor responsible for the hippocampus insult, but rather MV itself [13].

#### Neural signaling pathway

Two preclinical papers investigated the neural pathway for brain insult after MV [3, 18]. It has been proposed that the neural signal coming from the vagus nerve triggers neuroinflammation and brain injury during MV [3]. In the postulated neural pathway for VIBI, the vagal afferent signal changes as a result of cyclical alveolar stretch due to positive-pressure MV, leading to activation of pulmonary transient receptor potential vanilloid channel type 4 (TRPV4), and consequently to a reduction in gene expression of pulmonary TRPV4 and purinergic type 2X receptors [18]. According to the authors, the pulmonary TRPV4 activation would lead to a hippocampal overexpression of type 2 dopamine receptors, which would deactivate the B/glycogen synthetase kinase 3β (Akt/GSK3β), initiating the apoptotic cascade [18]. In order to demonstrate the hypothesized neural signaling pathway for VIBI, researchers compared hippocampal apoptosis in mechanically ventilated subjects with chemical vagotomy, with surgical vagotomy, and without vagotomy, and in never-ventilated subjects [3, 18]. It was found that vagotomy, either chemical or surgical, mitigated ventilation-induced brain injury [3]. The vagotomised groups had levels of hippocampal apoptosis similar to the never-ventilated group, resulting in the conclusion that the vagal signal triggered the brain injury after the initiation of MV [3, 18]. Moreover, to demonstrate that dopamine overexpression was part of the mechanism that triggered cellular apoptosis, the authors showed that the administration of a dopamine blocker mitigated the brain insult after MV in a group of subjects without vagotomy, under the same conditions that led to brain insult in mechanically ventilated subjects [3].

### Cognitive impairment and MV (preclinical)

Cognition after MV was evaluated in three preclinical studies identified in our search [5, 6, 14]. Two papers demonstrated that experimental mice undergoing six hours of MV had lower cognitive scores at three days post-extubation than either one-hour-MV mice or never-ventilated mice [5, 6]. One paper showed that a possible mechanism for cognitive impairment was the overexpression of TLR4 receptors in the lungs and in the brain, triggering inflammation and promoting the proliferation of pro-inflammatory microglia and reactive astrocytes, impairing brain function [6]. To demonstrate the role of neuroinflammation in cognitive impairment, the authors showed that TLR4-knockout subjects undergoing prolonged MV had similar microglia, reactive astrocytes, systemic inflammatory markers, and cognitive scores to control subjects [6]. Moreover, in prolonged mechanically ventilated subjects, neuroinflammation resulted in synapse degeneration, cytochrome c release, cleaved caspase-3, and cleaved PARP-1 activation, which may have consequently led to the worse cognitive scores observed in this group compared to the control group [5]. The effects of inflammation on cognition were assessed in one paper [14]; mechanically ventilated pigs with hypoxemia caused by lung injury due to surfactant depletion had worse cognitive performance 5 days after extubation than mechanically ventilated pigs with hypoxemia caused by reduction in inspired oxygen concentration [14]. The authors concluded that the inflammatory process may be a key factor for cognitive impairment in pigs [14].

### The connection between tidal volume and brain activity

The connection between hippocampal activity and the breathing cycle was demonstrated by one preclinical study [18]. This study used functional MRI to analyze hippocampus activity, comparing higher-tidal-volume subjects with lower-tidal-volume subjects [18]. The authors demonstrated that higher-tidal-volume MV resulted in more hippocampus activity, and that higher activation of the hippocampus during high-tidal-volume MV was correlated with more tissue injury [18]. In addition to the hippocampus, other brain areas were also studied during MV. Greater numbers of c-Fos-positive cells were observed in the retrosplenial cortex and in the thalamus of high-tidal-volume subjects when compared to low-tidal-volume subjects [16]. C-Fos is a neuronal activity marker expressed after neuronal depolarization [40]. Neurons express c-Fos protein proportionally to the stimulus applied, either chemical or electrical [40]. In low-tidal-volume subjects, c-Fos was expressed at low levels, while in high-tidal-volume subjects, this neuronal activity marker was expressed at high levels [16]. The authors stated that the tidal volume used during MV may have led to pathological neuronal activity in the retrosplenial cortex and in the thalamus, since the high-tidal-volume group expressed greater c-Fos protein in these brain areas than the low-tidal-volume group [16]; also, the brain insult observed was proportional to the tidal volume delivered, suggesting a potential iatrogenic effect of MV on the brain [16].

### Current clinical literature perspective on cognitive impairment and MV

Mechanically ventilated patients are frequently sedated and typically have worse health conditions than patients who are never ventilated [19, 21, 22, 35, 37, 45]. It is extremely challenging to show any causative linkage between MV, delirium and cognitive impairment. This is in part because the MV “package” has multiple inseparable variables, such as sedation and physical immobility. For instance, mechanically ventilated patients receive more drugs and are more physically inactive than spontaneously breathing patients [19, 21, 22, 35, 37, 45]. The greater use of drugs and greater prevalence of physical inactivity in mechanically ventilated patients might be factors that also affect the health of the patient, worsening the cognitive functions [19, 21, 22, 35, 37, 45]. Additionally, widespread use of MV in a heterogeneous patient population makes the isolation of causal relationships difficult. Although multiple risk factors have been identified for delirium and long-term cognitive impairment in our systematic review, papers that utilized multivariate analysis have consistently shown either duration of MV as an independent variable associated with delirium, or delirium as an independent variable associated with prolonged duration of MV [16, 17, 20–29, 45]. Moreover, delirium in mechanically ventilated patients was correlated with a greater likelihood for long-term cognitive impairment than mechanically ventilated patients without delirium [20, 24, 26, 30, 38, 42]. The papers analyzed in this systematic review did not establish a direct causal link between duration of MV, delirium, and long-term cognitive impairment; however, our review identified important gaps in the literature that can be used in designing future studies.

### Duration of MV as an independent variable for developing delirium during hospitalization

Our systematic review identified twelve papers that showed an association between longer duration of MV and a greater likelihood of a patient developing delirium during hospitalization, when compared either with patients mechanically ventilated fewer days, or with spontaneously breathing patients [19–21, 24, 30, 32, 35, 36, 43–45]. For instance, ten papers found odds ratios between 1.06 and 10.50 for a greater likelihood that a patient develops delirium during hospitalization when the duration of MV is longer than one day (Fig. 2) [19–21, 24, 32, 35, 36, 43, 44]. Ten of these twelve papers showed a dose-dependent aspect, as, regardless of comorbidities, the longer the duration of MV, the greater the likelihood of delirium with a pooled odds ratio of 3.42 [19–21, 24, 30, 32, 35, 36, 43, 44]. No heterogeneity was observed after analysis of these ten papers. The homogeneity of these papers may be interpreted as a strong and consistent signal indicating that increased duration of MV increases the likelihood for delirium [19–21, 24, 30, 32, 35, 36, 43, 44].

### Delirium during hospitalization as an independent variable for prolonged duration of MV

Although multiple risk factors may prolong the days on MV in critically ill patients, delirium has been commonly identified as one of those risk factors for prolonged duration of MV [22, 25–27, 29, 31, 35, 36, 41]. Nine clinical papers found an association between delirium and prolonged MV, of which seven calculated the odds ratio and two calculated how much longer, either in days or hours, delirium prolonged MV [22, 25–27, 29, 31, 35, 36, 41]. Seven of nine papers identified by our systematic review showed that patients with delirium during hospitalization have greater likelihood to be mechanically ventilated longer than patients without delirium during hospitalization with a pooled odds ratio of 2.06 (Fig. 3) [22, 25, 27, 29, 31, 35, 36]. Patients who were diagnosed with delirium during hospitalization underwent between seven and ten more days on MV, compared with patients who were not diagnosed with delirium [35, 41]. Although a positive correlation between delirium and MV has been shown in our systematic review, the clinical literature has not reported any causative linkage between them [22, 25, 27, 29, 31, 35, 36]. Our systematic review indicates a high heterogeneity for the seven papers selected in this subgroup analysis. This may be a consequence of a small standard error for each of the studies included in the analysis. Another reason may be the high degree of heterogeneity typically observed in the ICU patient population studied resulting in a wide range of results reported. Different methods to measure the outcomes, different study designs and different types of interventions may also have affected heterogeneity. However, all studies included in this part of our analysis investigated delirium as an independent factor for prolonged MV, showing ORs higher than 1. Regardless of the heterogeneity of the papers analyzed, it seems that when an ICU patient develops delirium it increases the likelihood for prolonged MV.

### Delirium in mechanically ventilated patients associated with increased likelihood of long-term cognitive impairment

Delirium in mechanically ventilated patients was also found to be one risk factor associated with long-term cognitive impairment [20, 26, 38, 42]. Four papers included only mechanically ventilated patients, reported that patients who developed delirium during hospitalization had a greater likelihood of showing long-term cognitive dysfunction than patients who did not develop delirium during hospitalization, reporting odds ratios ranging from 3.20 to 7.86 with a pooled odds ratio of 3.76 (Fig. 4) [20, 26, 38, 42]. Moreover, in mechanically ventilated patients with delirium during hospitalization, long-term cognitive deficits were subsequently identified up to seven times as often, compared to mechanically ventilated patients without delirium during hospitalization, although this may be due to underlying predisposition rather than MV itself [20, 26, 38, 42]. These four papers reported that more than 2 days of MV is associated with up to four times greater likelihood of developing acute cognitive impairment, and that those patients who develop acute cognitive impairment have up to twice the risk of persistent chronic cognitive impairment [20, 26, 30, 38, 42]. The residual impact of delirium in mechanically ventilated patients was detected up to 6 years after hospital discharge [20, 42]. The lack of heterogeneity observed may be an indicator that when a mechanically ventilated patient develops delirium it considerably increases the likelihood for long-term cognitive impairment.

### Gaps in the current literature and the need for future studies

Our systematic review identified some notable gaps in the scientific literature. Firstly, we note that none of the identified preclinical or clinical papers investigated ventilatory strategies, focusing, for example, either on ventilation power or on driving pressure, and their effects on cognitive outcomes. Three preclinical papers investigated alternative methods, either pharmacological or surgical, to prevent VIBI [3, 6, 18]. Dopamine receptors, TLR-4 receptors and TRPV4 receptors are pharmacological targets that, when blocked, were reported to prevent VIBI. In addition, either chemical or surgical vagotomy also showed reduced hippocampal apoptosis and inflammation, even during high-tidal-volume MV; however, vagotomy is not a viable solution in clinical practice [3, 6, 18]. Secondly, only nine preclinical publications were identified in our search; this suggests that more work is needed in this area in order to better understand the effects of MV on the brain [3, 5, 6, 13–18]. Notably, all nine preclinical studies used injurious (high-tidal-volume or high peak-inspiratory-pressure) ventilation; this observation raises the question as to whether the effects of lung-protective MV on the brain should also be studied preclinically [3, 5, 6, 13–18]. Thirdly, of the preclinical studies identified, three observed greater neuronal activity during MV; this finding should be more thoroughly investigated in future studies to better understand whether the changes in the neurophysiology during MV result in harmful effects on the brain [16]. Fourthly, it is important to recognize that many other factors are linked to delirium and cognitive impairment in critically ill patients; our systematic review found that MV may be associated with delirium, but no study showed any causative linkage between MV and delirium. Considering these observations, more preclinical studies should be designed focusing on the investigation of potential causal links between MV, brain insult, and cognitive impairment, and more clinical studies should be designed to investigate the possibility of causal links between MV, brain insult, and delirium and cognitive impairment, controlling for potentially confounding factors that co-vary with duration of MV, such as sedation and immobility.

## Conclusion

This systematic review showed an association between MV and acute cognitive impairment.

In our search, preclinical papers showed acute cognitive impairment after MV, describing greater neuroinflammation and lower cognitive scores in subjects with longer duration of MV.

Clinically, increased duration of MV may be associated with a greater risk for delirium during hospitalization. Moreover, delirium in mechanically ventilated patients may be associated with long-term cognitive impairment, and residual cognitive impairment can be observed up to 6 years after hospital discharge.

Preclinical and clinical studies that investigate the relationship between different ventilation strategies and cognitive impairment have not been reported. Conducting such studies may be worthwhile in order to better understand cognitive impairment after MV.

While our systematic review identified gaps in the literature that can be considered when designing future studies to further evaluate the relationships between MV, brain insult, and cognitive impairment, our findings confirm that future work is needed to identify any causal links between them.

## Supplementary Information

**Additional file 1.** Additional information about the systematic review, such as keywords used for the search and exclusion criteria used.

## Data Availability

The datasets used and/or analyzed during the current study are available from the corresponding author on reasonable request.
