# Supplementary material for: Systematic review of cognitive impairment and brain insult after mechanical ventilation
Source: Crit Care. 2021 Mar 10;25:99. doi: 10.1186/s13054-021-03521-9 (PMC7945325; doi:10.1186/s13054-021-03521-9)
Supplement: Supplementary file 1 — Additional file 1. Additional information about the systematic review, such as keywords used for the search and exclusion criteria used. [file 13054_2021_3521_MOESM1_ESM.docx]

**Title:  Systematic review of cognitive impairment and brain insult after mechanical ventilation. (89 characters)**

Thiago G. Bassi^1,2^, Elizabeth C. Rohrs^1,3^, Steven C. Reynolds^1,3^

Table A. Preclinical and clinical key words

| Preclinical Key Words | Clinical Key Words |
| --- | --- |
| 1. Respiration, artificial | 1. Mechanical ventilation |
| 1. Mechanical ventilation | 1. Cognitive dysfunction |
| 1. Cognitive dysfunction | 1. Neurological injury |
| 1. Neurological injury | 1. Neurological complication |
| 1. Neurological complication | 1. Neurological harm |
| 1. Neurological harm | 1. Neuropsychological injury |
| 1. Neuropsychological injury | 1. Neuropsychological complication |
| 1. Neuropsychological complication | 1. Neuropsychological harm |
| 1. Neuropsychological harm | 1. Neurocognitive injury |
| 1. Neurocognitive injury | 1. Neurocognitive complication |
| 1. Neurocognitive complication | 1. Neurocognitive harm |
| 1. Neurocognitive harm | 1. Neurological |
| 1. Neurological | 1. Neuropsychological |
| 1. Neuropsychological | 1. Neurocognitive |
| 1. Neurocognitive | 1. Delirium |
| 1. Apoptosis | 1. ----- |
| 1. Caspase 8 | 1. ----- |
| 1. Caspase 9 | 1. ----- |
| 1. Glycogen synthase kinase 3 beta | 1. ----- |
| 1. Ventilator-induced brain injury | 1. ----- |
| 1. Dysbindin | 1. ----- |
| 1. Lung-brain interaction | 1. ----- |
| 1. Vagus nerve | 1. ----- |
| 1. Receptors, dopamine | 1. ----- |
| 1. Hippocampal | 1. ----- |
| 1. Learning disorders | 1. ----- |
| 1. Memory disorders | 1. ----- |

Table B. Preclinical and clinical exclusion criteria

| Preclinical Exclusion Criteria | Clinical Exclusion Criteria |
| --- | --- |
| 1. Articles in languages other than English | 1. Articles in languages other than English |
| 1. Articles that are clearly not relevant to the review objective | 1. Articles that are clearly not relevant to the review objective |
| 1. Articles about human research | 1. Articles about animal or in vitro research |
| 1. Reviews, comments, editorials, letters to the editor | 1. Reviews, comments, editorials, letters to the editor |
| 1. Single case reports and case series with less than 6 subjects in total | 1. Single case reports and case series with less than 10 patients in total |
| 1. Studies focused on animal neuro-interventions intended to injure the central nervous system causing cardiac arrest, traumatic brain injury, stroke, SAH, or neurodegenerative diseases | 1. Studies focused on patients admitted to the ICU because of cardiac arrest, traumatic brain injury, stroke, SAH, neurodegenerative diseases, brain cancer, or cardiac surgery history |
| 1. **---** | 1. Articles concerning pediatrics (newborns/infants, children, adolescents) |
